# Nitric Oxide Turnover Under Hypoxia Results in the Rapid Increased Expression of the Plastid-Localized Phosphorylated Pathway of Serine Biosynthesis

**DOI:** 10.3389/fpls.2021.780842

**Published:** 2022-01-31

**Authors:** Somaieh Zafari, Greg C. Vanlerberghe, Abir U. Igamberdiev

**Affiliations:** ^1^Department of Biology, Memorial University of Newfoundland, St. John’s, NL, Canada; ^2^Department of Biological Sciences, University of Toronto Scarborough, Toronto, ON, Canada; ^3^Department of Cell and Systems Biology, University of Toronto Scarborough, Toronto, ON, Canada

**Keywords:** *Nicotiana tabacum*, alternative oxidase, γ-aminobutyric acid shunt, class 1 phytoglobin, hypoxic stress, nitric oxide, phosphorylated pathway of serine biosynthesis

## Abstract

The plant mitochondrial electron transport chain influences carbon and nitrogen metabolism under near anoxic conditions through its involvement in the phytoglobin-nitric oxide cycle, where the respiratory chain reduces nitrite to nitric oxide (NO), followed by NO conversion to nitrate by class 1 phytoglobin. Wild type (WT) and transgenic tobacco (*Nicotiana tabacum* L.) with differing amounts of alternative oxidase (AOX) were used to manipulate NO generation under hypoxia, and to examine whether this in turn influenced the gene expression of two stress-related amino acid biosynthetic pathways, the plastid-localized phosphorylated pathway of serine biosynthesis (PPSB), and the γ-aminobutyric acid (GABA) shunt. Under hypoxia, leaf NO emission rate was highest in AOX overexpressors and lowest in AOX knockdowns, with WT showing an intermediate rate. In turn, the rate of NO emission correlated with the degree to which amino acids accumulated. This amino acid accumulation was associated with the increased expression of the enzymes of the stress-related amino acid biosynthetic pathways. However, induction of the PPSB occurred much earlier than the GABA shunt. This work shows that high rates of NO turnover associate with rapid gene induction of the PPSB, establishing a clear link between this pathway and the maintenance of carbon, nitrogen and energy metabolism under hypoxia.

## Introduction

Plant acclimation to oxygen deficiency involves increased glycolytic fermentation (Kennedy et al., 1992) and upregulation of nitrate reductase (NR) (Botrel and Kaiser, 1997). The latter relates to nitrogen turnover by the class 1 phytoglobin-nitric oxide (Pgb1-NO) cycle, an alternative to the classical fermentation pathways (Igamberdiev and Hill, 2004, 2018; Gupta and Igamberdiev, 2011; Hebelstrup and Møller, 2015). In this cycle, the mitochondrial electron transport chain (mETC) reduces nitrite to NO, Pgb1 converts the NO to nitrate, and NR converts the nitrate back to nitrite. The mETC activity associated with this cycle allows for limited ATP production when oxygen is limiting.

In addition to cytochrome c oxidase, the plant mETC includes a second terminal oxidase, an ubiquinol oxidase termed alternative oxidase (AOX) (Selinski et al., 2018; Vanlerberghe et al., 2020). Under normoxic conditions, AOX activity can dampen NO generation by preventing the over-reduction of mETC components (Cvetkovska and Vanlerberghe, 2012; Gupta et al., 2014; Alber et al., 2017). However, under hypoxic (near anoxic) conditions, AOX activity accelerates NO generation. This conclusion is based on chemical inhibitor studies (Tischner et al., 2004; Planchet et al., 2005) and studies using AOX knockout/knockdown and overexpression plants of Arabidopsis thaliana (Vishwakarma et al., 2018) and *Nicotiana tabacum* (Jayawardhane et al., 2020). The AOX protein contains non-heme iron, making possible its involvement in various redox reactions. Nonetheless, a direct demonstration of the catalysis of nitrite to NO by AOX remains lacking. Hence, it is possible that the effect of AOX on NO metabolism under hypoxia is by some indirect mechanism, as discussed elsewhere (Jayawardhane et al., 2020).

Studies have shown that hypoxia can result in the accumulation of amino acids such as alanine and γ-aminobutyric acid (GABA) (Miyashita and Good, 2008; Rocha et al., 2010; Mustroph et al., 2014; António et al., 2016; Wu et al., 2021). This likely reflects specific interactions of carbon and nitrogen metabolism to support energy metabolism under hypoxia. For example, GABA accumulation may contribute to the maintenance of cytosolic pH (Crawford et al., 1994; Bown and Shelp, 2016; Igamberdiev and Hill, 2018). Interestingly, the profile of amino acids accumulating under hypoxia may differ depending upon AOX amount (Gupta et al., 2012; Jayawardhane et al., 2020). This hints that the Pgb1-NO cycle, whose function also depends upon AOX amount, may influence the specific pathways of carbon and nitrogen metabolism that result in amino acid accumulation under hypoxia.

This study used wild type (WT) and transgenic plants with differing amounts of AOX to manipulate the rate of NO generation under hypoxia. The rate of NO generation effected the gene expression of enzymes involved in NO turnover, as well as the energy status and amino acid pool size of the tissue under hypoxia. Further, high rates of NO turnover resulted in the rapid induction of the plastid-localized “phosphorylated pathway of serine biosynthesis” (PPSB) (Ros et al., 2014). This reveals a previously unrecognized role of the PPSB in carbon and nitrogen metabolism under hypoxia.

## Materials and Methods

### Plant Material, Growth Condition, and Hypoxia Treatment

All experiments included wild type (WT) tobacco (*Nicotiana tabacum* L. cv. Petit Havana SR1), two AOX overexpression lines (B7, B8), and two AOX knockdown lines (RI9, RI29), all of which are previously described (Wang et al., 2011; Wang and Vanlerberghe, 2013). Tobacco wild type (WT), AOX knockdown and overexpression plants were grown in controlled-environment growth chambers for 4 weeks prior to experiments, as previously described (Jayawardhane et al., 2020). To gain a comprehensive insight into how AOX interconnects to the PPSB and GABA shunt under low oxygen, plants were subjected to nitrogen atmosphere for an extensive time course, and the samples were taken at 0 h (normoxia), 3, 6, 12, 24, and 48 h of the anoxic condition. For experimentation, the plants were placed in a custom-built, sealed, and dark chamber, to which the air supply could be replaced by nitrogen gas containing 0.001% oxygen (Cochrane et al., 2017). This treatment limits both aerobic respiration and photosynthesis by depriving plants of oxygen and light. The plants endure a progressive decrease in oxygen availability and some cells may reach a state of anoxia. The control plants were treated with normal air in the same chamber. After treatment, the fourth leaf from the top of each plant was removed, quickly frozen in liquid nitrogen, and stored at −80°C. The images of overexpressors B8 and B7, WT, and knockdowns RI9 and RI29 under normoxic conditions and after anoxic treatment were provided in the earlier study (Jayawardhane et al., 2020).

### Nitric Oxide Emission

Gas phase NO was measured by the chemiluminescence detection method as described (Planchet et al., 2005). The leaves were detached from the plants and placed in 20 mM Hepes buffer (pH 7.0) with 50 mM sodium nitrate as nitrogen source. The leaves in the buffer were immediately placed in glass chamber with a continuous constant inflow of nitrogen at 120 mL min^–1^. The air-tight chamber was filled with measuring gas (purified air or nitrogen) which was pulled through the chemiluminescence detector (CLD 88 p; Eco-Physics, Dürnten, Switzerland) by vacuum pump connected to ozone destroyer. The measuring gas was made NO free by a NO scrubber supplied by Eco Physics Ltd., Switzerland. Chemiluminescence analyzer was calibrated daily with NO-free air and with various NO concentrations (1–35 ppb) adjusted by mixing the calibration gas (500 ppb NO in nitrogen, Messer Griesheim, Darmstadt, Germany) with NO-free air. Flow controllers (Fisher Scientific) were used to adjust all gas flows.

### Metabolites

About 100 mg of fresh frozen biomass were immediately gently homogenized in 1 mL of 2.4 M perchloric acid. The homogenate was then neutralized using 5 M KOH and centrifuged at 16,000 × *g* for 10 min at 4°C (Dordas et al., 2003). The ADP/ATP ratio was measured by luciferase-based assay kit (Enzylight™ ADP/ATP ratio assay kit; BioAssay Systems, Hayward, CA, United States) on FB 12 Single Tube Luminometer (Berthold Detection Systems, GmbH, Germany).

The content of free amino acids was calculated using the approach of Lee and Takahashi (1966). 0.5 g of plant material was extracted by 70% ethanol, incubated overnight and then centrifuged at 14,000 × *g* for 15 min at 4°C. 1 mL extract was mixed with 0.5 M citrate buffer (pH 5.6), 55% glycerol, and ninhydrin solution, and then heated at 100°C for 30 min. The test tubes were immediately chilled and gently shaken. The absorbance at 570 nm was measured using a spectrophotometer. Glycine in citrate buffer was used as a standard.

### Enzyme Assays

Nitrate reductase (NR, EC 1.7.1.1-3) activity of tobacco leaves was measured by the method of Ferrario-Mery et al. (1998). The activity was assayed in the reaction mixture consisting of 50 mM HEPES-KOH, pH 7.5, 0.1 mM NADH, 5 mM KNO_3_, and 2 mM EDTA. After 15 min, the reaction was stopped by adding equal volumes of 1% sulfanilamide and 0.02% naphthylethylene-diamine dihydrochloride and nitrite concentration was determined at 540 nm.

Phosphoglycerate kinase (PGK, EC 2.7.2.3) activity was evaluated by measuring NADH oxidation, in a coupled reaction with glyceraldehyde phosphate dehydrogenase (GAPDH) (Rosa-Téllez et al., 2018). The activity was assayed in the reaction mixture containing 100 mM HEPES-KOH, 1 mM EDTA, 2 mM MgSO_4_, 0.3 mM NADH, 6.5 mM PGA, 1 mM ATP, and 3.3 units of GAPDH. The reaction was initiated by adding protein extracts and the absorbance was read at 340 nm.

### Transcript Amounts

RNA was extracted from frozen leaf using the FastRNA^®^ Pro Green Kit (MP Biomedicals), according to the manufacturer’s instructions. RNA (5 μg) was reverse transcribed using the Superscript III reverse transcriptase kit (Invitrogen). Quantitative PCR was carried out with an Applied Biosystems StepOnePlus Real-Time PCR System, using SYBR Green qPCR Master Mixes (Thermo Scientific) and gene-specific primers (Table 1). Comparative quantification was performed by the ΔΔC_*t*_ method, with actin as the reference gene. All transcript amounts are relative to the amount in WT at time 0 (log2 fold-change). For a subset of genes (those encoding PGDH and PSP) we also confirmed the gene expression patterns using a second reference gene encoding GAPDH.

**TABLE 1:** Primers used for qRT-PCR.

Gene/protein	Forward	Reverse	Accession #
Actin	TTGGCTTACATTGCTCTTG	TCATTGATGGTTGGAACAG	XM_033660572.1
PGDH	GGCCTCGGTATGCATGTAAT	CCTGCTCAAAGGAAACCAAATC	XM_009612696.3
PSAT	CCACCCTCACCTCTACCTATAA	GAAGTTGAAGACCCGATCTGAG	XM_016597132.1
PSP	CTGGAAAGGCTGTAGCAGAA	CCTGGAGTTGGGACAATGAA	XM_016601311.1
GABA-T	AGTCCCATAGTCGGAGAGATTAG	GGGAAAGGATCATTGGGAGATT	NM_001324662.1
GAD	TGAAGTGGAGCTAAAGGAAGTAAA	TTGAGTGTGGAACCCAAGATAG	U54774.1
Pgb1	TGCCAAACTCAAGCCTCAT	TCGAATCCCTCACCACTACT	KJ808726.1
GSNOR	TCTGCCACACTGATGCTTATAC	CTCCTTCACCGACACTTTCTAC	XM_009631265
NR	CACTCGAGGTTGAGGTGTTAG	AGCTTCTCGGGTTGAGTATTG	X14059.1
GAPDH	GGCAGCATCCTTCAACATTATTC	CTCGGAAAGCCATTCCAGTTA	KR007670.1

*PGDH, phosphoglycerate dehydrogenase; PSAT, phosphoserine aminotransferase; PSP, phosphoserine phosphatase; GABA-T, GABA transaminase; GAD, glutamate decarboxylase; Pgb1, class 1 phytoglobin; GSNOR, S-nitrosoglutathione reductase; NR, nitrate reductase; GAPDH, glyceraldehyde phosphate dehydrogenase.*

### Statistical Analysis

All experiments were repeated three times. To statistically analyze the data of tobacco leaves, the software package SPSS V. 21.0 (Statistical Package for Social Science; Chicago, IL, United States) was used. To determine significant differences between the WT, AOX overexpressors, and AOX knockdowns, one-way ANOVA was conducted by using Duncan’s multiple range. Data in the text and figures are presented as mean value ± standard deviations of three replicates. The differences with *p* ≤ 0.05 were considered as statistically significant. Only statistically significant differences are discussed in the manuscript.

## Results

### Nitric Oxide Emission, Energy State, and Amino Acid Content of Plants Under Hypoxia

**FIGURE 1:**
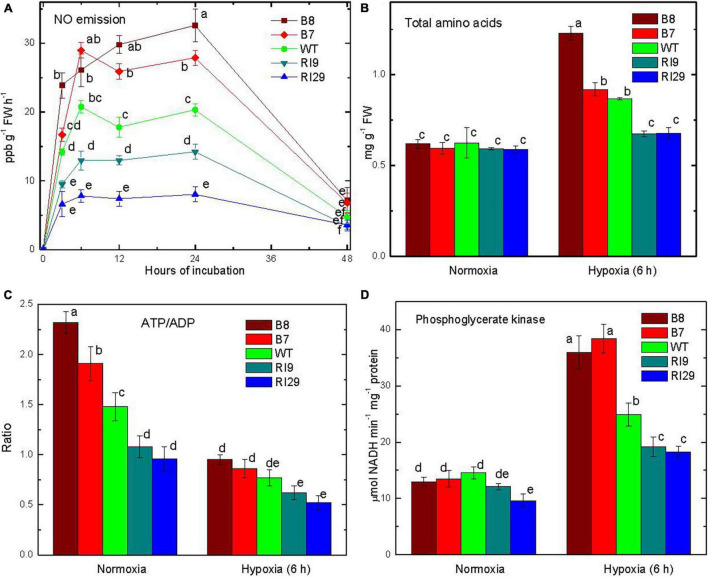
Leaf nitric oxide emission **(A)**, total amino acid content **(B)**, ATP/ADP ratio **(C)**, and phosphoglycerate kinase activity **(D)** in tobacco plants with differing amounts of alternative oxidase, and exposed to hypoxia (at time 0 in **A**). The plant lines used included wild type (WT), two alternative oxidase overexpressors (B7, B8), and two alternative oxidase knockdowns (RI9, RI29). Vertical bars indicate SD from three to four independent experiments (*n* = 3–4); different letters indicate significant differences between five tobacco lines and the time points.

Tobacco leaves in a hypoxic (near anoxic, ∼0.001% O_2_) atmosphere emitted NO, while no emission was detected from leaves in air (Figure 1A). The NO emission rate depended upon AOX amount. Rates were 50% higher in AOX overexpressors (B7, B8) than WT throughout the first 24 h of hypoxia. The AOX knockdown lines (RI9, RI29) had much lower NO emission rates than WT. In all plant lines, NO emission rates declined by 48 h of hypoxia, perhaps due to the poor physical condition of the plants by this time point (Figure 1A).

In air, all of the plant lines had a similar total pool size of amino acids in the leaf (Figure 1B). The pool size of amino acids increased following 6 h of hypoxia, and was now highest in the overexpressors (particularly B8) and lowest in the knockdowns, with WT showing an intermediate amount. In air, the leaf ATP/ADP ratio was higher by 1.5 times in the overexpressor B8 and lower by more than 50% in the knockdown RI29 as compared to WT (Figure 1C). Overall, ATP/ADP ratios decreased following 6 h hypoxia, but the relative differences across plant lines persisted. In hypoxia, B8 had 1.2-fold greater ATP/ADP ratio than WT, while RI29 had a 1.5-fold lower ratio. In air, the maximum PGK activity, which represents a measure of the glycolytic flux capacity (Rosa-Téllez et al., 2018), was similar across plant lines (Figure 1D). The PGK activity increased in all plant lines following 6 h of hypoxia. However, the activity was now highest in the overexpressors and lowest in the knockdowns, with WT showing an intermediate activity (Figure 1D).

### Components of Nitric Oxide Turnover

**FIGURE 2:**
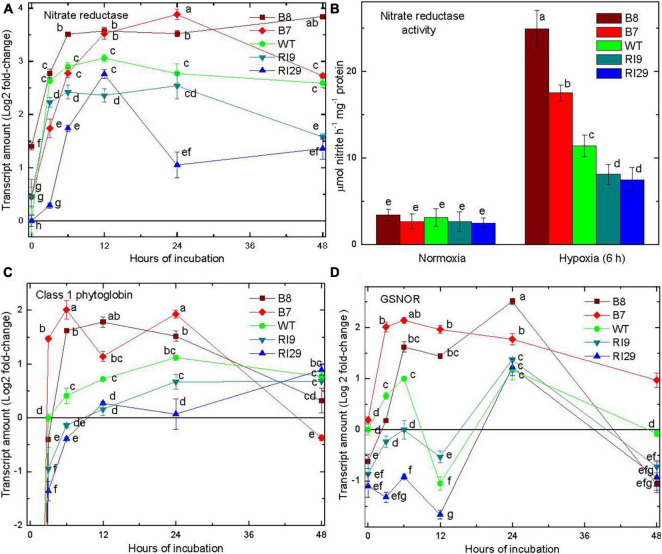
Leaf nitrate reductase transcript amount **(A)**, nitrate reductase activity **(B)**, class 1 phytoglobin transcript amount **(C)**, and *S*-nitrosoglutathione reductase transcript amount **(D)** in tobacco plants with variable amounts of alternative oxidase, and exposed to hypoxia (at time 0 in **A,C,D)**. In **(C)**, there was no detectable transcript at time 0. Vertical bars indicate SD from three independent experiments (*n* = 3); different letters indicate significant differences between five tobacco lines and the time points.

NR transcript amount increased rapidly in all plant lines in response to hypoxia (Figure 2A). NR activity showed no significant changes across plant lines under normal conditions. The activity increased substantially in response to a 6 h hypoxia treatment and was now highest in the overexpressors and lowest in the knockdowns, with WT showing an intermediate activity (Figure 2B). The overexpressor line B8 had a 2.1- and 3.3-fold higher activity than knockdowns, RI9 and RI29, respectively.

In air, there was no detection of the transcript encoding Pgb1 in any plant line. However, this transcript was readily detectable following 3 h of hypoxia (Figure 2C). Further, the transcript amounts over the first 24 h under hypoxia were highest in the overexpressors and lowest in the knockdowns, with WT showing an intermediate amount. By 48 h, transcript amounts declined and were similar across plant lines (Figure 2C).

Following 6 h of hypoxia, the transcript amount of *S*-nitrosoglutathione reductase (GSNOR) was highest in the overexpressors and lowest in the knockdowns, with WT showing an intermediate amount (Figure 2D). Interestingly, the WT and knockdown lines displayed a clear oscillation of transcript amount (high at 6 h, lower at 12 h, up again by 24 h) that was not evident in the overexpressors, where transcript amount remained high through 24 h. All plant lines showed a decline in GSNOR transcript amount between 24 and 48 h (Figure 2D).

### The Phosphorylated Pathway of Serine Biosynthesis

**FIGURE 3:**
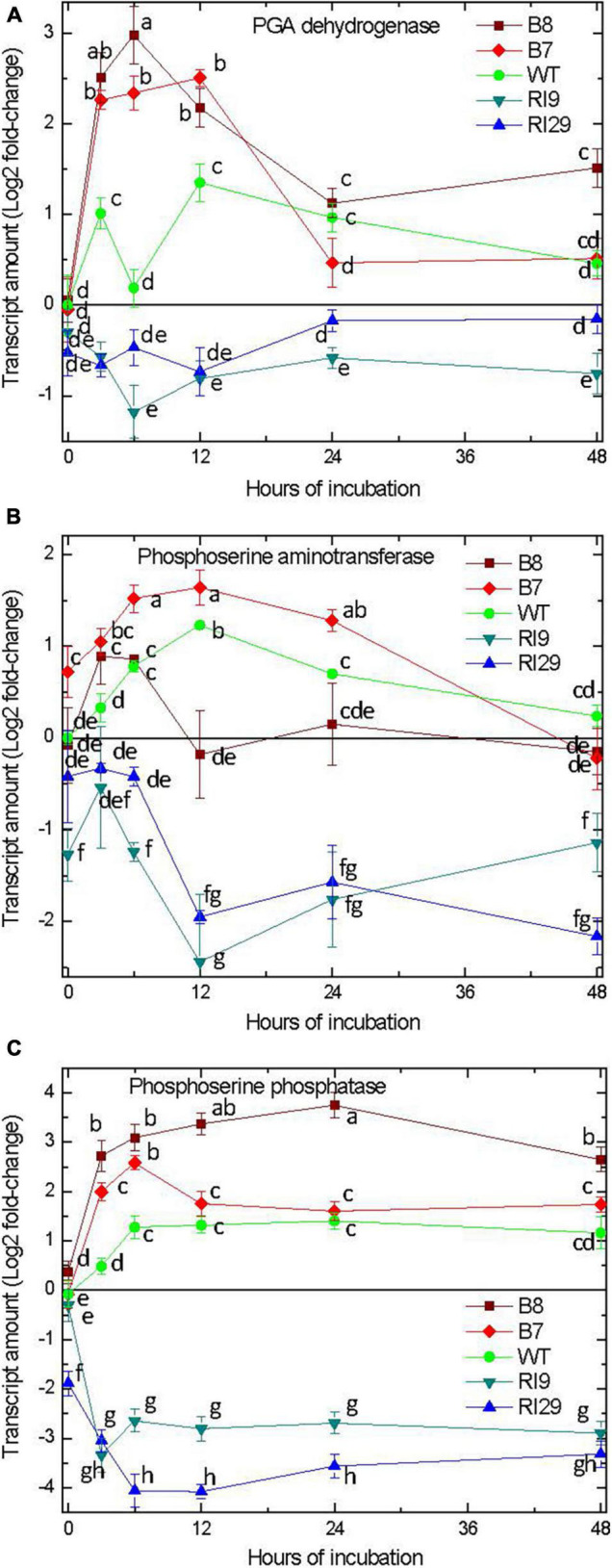
Leaf transcript amount for enzymes of the phosphorylated pathway of serine biosynthesis, including 3-phosphoglycerate dehydrogenase **(A)**, 3-phosphoserine aminotransferase **(B)**, and 3-phosphoserine phosphatase **(C)** in tobacco plants with differing amounts of alternative oxidase and exposed to hypoxia at time 0. For 3-phosphoglycerate dehydrogenase **(A)** and 3-phosphoserine phosphatase **(C)** the normalized transcript levels are shown using two reference genes, actin and GAPDH. Vertical bars indicate SD from three independent experiments (*n* = 3); different letters indicate significant differences between five tobacco lines and the time points.

We examined the gene expression of enzymes within the plastid-localized PPSB (Ros et al., 2014). In response to hypoxia, the transcript amounts for 3-phosphoglycerate dehydrogenase (PGDH), 3-phosphoserine aminotransferase (PSAT), and 3-phosphoserine phosphatase (PSP) increased in the WT and AOX overexpression plants, with the overexpressors clearly showing the greatest increases (Figure 3). Depending upon the plant line and enzyme, transcript abundance peaked between 3 and 12 h of hypoxia treatment. Thereafter, transcript amount either remained steady (PSP) or gradually declined (PGDH, PSAT). The knockdown plants acted very differently. In this case, the hypoxia treatment either had no effect on transcript amount (PGDH) or resulted in declines in transcript amount (PSAT, PSP) (Figure 3). The results of PGDH and PSAT expression obtained by using the second reference gene encoding GAPDH were quite similar confirming the results with the actin reference gene. The data for the transcript amounts of PGDH and PSAT are shown with actin and GAPDH as the reference genes (Figures 3A,C).

### The γ-Aminobutyric Acid Shunt

**FIGURE 4:**
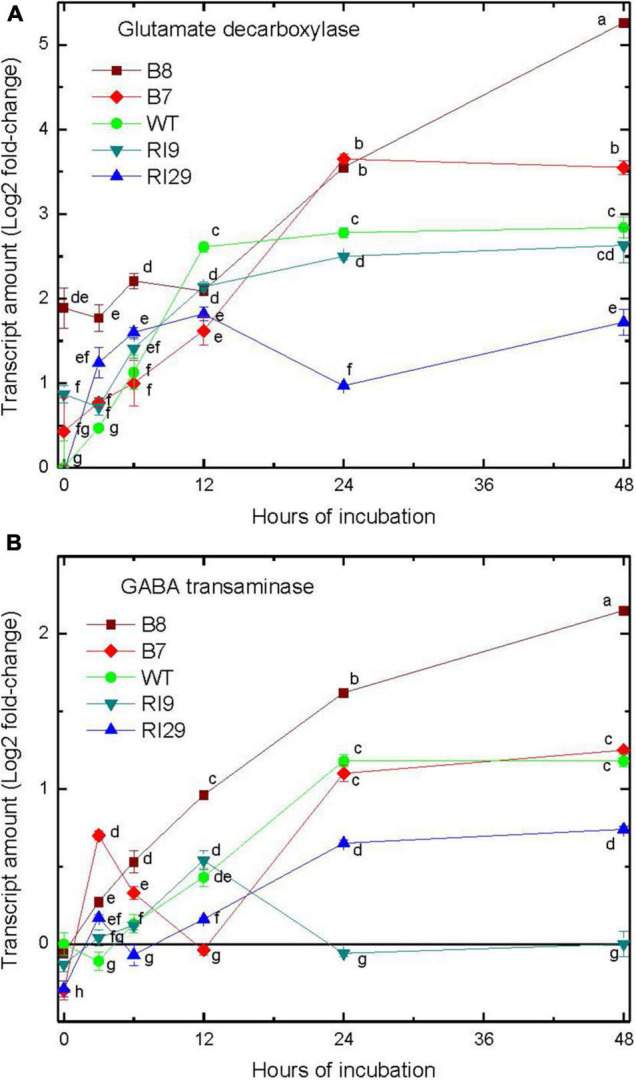
Leaf transcript amount for enzymes of the γ-aminobutyric acid shunt, including glutamate decarboxylase **(A)** and γ-aminobutyrate transaminase **(B)** in tobacco plants with differing amounts of alternative oxidase, and exposed to hypoxia at time 0. Vertical bars indicate SD from three independent experiments (*n* = 3); different letters indicate significant differences between five tobacco lines and the time points.

We examined the gene expression of enzymes within the GABA shunt, including glutamate decarboxylase (GAD; responsible for GABA synthesis), and γ-aminobutyrate transaminase (GABA-T; catalyzing conversion of GABA to succinic semialdehyde) (Bown and Shelp, 1997). The abundance of these transcripts did increase in response to hypoxia, but with an obvious lag compared to the transcripts associated with the PPSB (Figure 4). In the case of these GABA shunt enzymes, transcript increases were relatively moderate within the first 12 h of hypoxia, and without any clear differences in abundance across the plant lines. However, following 24–48 h of hypoxia, there were clear increases in transcript abundance, and transcript amounts were highest in the overexpressors and lowest in the knockdowns (Figure 4).

## Discussion

### Interaction of Alternative Oxidase and the Pgb1-Nitric Oxide Cycle in Energy Metabolism Under Hypoxia

AOX is a non-energy conserving ubiquinol oxidase of the plant mETC (Vanlerberghe et al., 2020). This study confirms previous work in *Arabidopsis* (Vishwakarma et al., 2018) and tobacco (Jayawardhane et al., 2020) that AOX amount is an important determinant of NO generation under hypoxia. Overexpression of AOX increased NO generation, while knockdown of AOX decreased NO generation, relative to WT. These differences in NO generation corresponded with differences in the expression/activity of key enzymes involved in the Pgb1-NO cycle including NR and Pgb1 (Figures 2A–C). In both cases, expression/activity were greatest in the plant lines with highest NO emission (AOX overexpressors) and lowest in the plant lines with lowest NO emission (AOX knockdowns). This is further evidence that the source of NO generation under hypoxia is the Pgb1-NO cycle (Jayawardhane et al., 2020). Interestingly, GSNOR expression also increased under hypoxia (Figure 2D). GSNOR can buffer against high tissue concentrations of NO by degrading *S*-nitrosoglutathione, a bioavailable cellular reservoir of NO (Sakamoto et al., 2002; Gupta et al., 2011; Li et al., 2021). The differential expression of this important player in NO metabolism across plant lines is another indication that AOX amount is an important determinant of NO generation under hypoxia (Jayawardhane et al., 2020).

An intriguing observation arising from an earlier study (Gupta et al., 2012) is that NR activity is required for increased AOX expression under hypoxia and from this study, it follows that vice versa AOX activity determines NR expression under hypoxia. This strongly supports the conclusion that AOX participates in the Pgb1-NO cycle and indirectly indicates that AOX may be involved in the production of NO from nitrite as suggested by Vishwakarma et al. (2018). While NR and Pgb1 are well known as essential components of the Pgb1-NO cycle (Igamberdiev et al., 2005), AOX appears to be another important factor, whose contribution may be comparable to that of other electron transport chain components.

The AOX-dependent changes in Pgb1-NO cycle activity under hypoxia impacted leaf energy status. Under normoxia, induction of AOX in response to diverse stressors can decrease electron transfer through complexes III and IV, preventing electron leakage to nitrite and consequent NO buildup (Cvetkovska and Vanlerberghe, 2012). Excess NO can block complex IV (lowering the ATP/ADP ratio); as a result, the AOX reduces nitrite-dependent NO synthesis (raising the ATP/ADP ratio) that would otherwise result from increased electron leakage in the cytochrome pathway. In fact, even under normoxia, AOX had a positive impact on energy status, despite its non-energy conserving nature. This indicates that tobacco AOX must optimize leaf respiration, perhaps by preventing bottlenecks in carbon metabolism and/or controlling the generation of reactive oxygen and nitrogen species (Cvetkovska and Vanlerberghe, 2012; Dahal and Vanlerberghe, 2017; Selinski et al., 2018; Jayawardhane et al., 2020). In contrast to its function under normal air, AOX has a unique role in hypoxia, when it can boost nitrite-dependent NO generation (Vishwakarma et al., 2018). This reaction drives the Pgb1-NO cycle to increase energy efficiency under hypoxia (Kumari et al., 2019).

The observation that the ATP/ADP ratio in AOX overexpressors substantially higher than in WT under normal conditions is also notable. One potential explanation is related to the amount of NO in these transgenic lines under normoxia and is due to prevention of overreduction of the components of the mitochondrial ETC, in particular at the level of one-electron leakage at the site of Complex III. The difficulty in comparing NO between the WT and overexpressors under normoxia is that it is very low in both, so a difference cannot be easily measured. It is known that NO has a strong inhibitory effect on cytochrome *c* oxidase. Lower levels of NO in the mitochondria of the overexpression lines may promote cytochrome oxidase activity and thus raise the ATP/ADP ratio, whereas greater levels of NO in the mitochondria of the knockdown lines under normoxia suppress cytochrome *c* oxidase and thus reduce the ATP/ADP ratio. Furthermore, it was previously demonstrated that reduced NO levels in barley roots caused by non-symbiotic phytoglobin overexpression increased respiration rates in normoxia (Gupta et al., 2014). We previously demonstrated that when hypoxic leaves were pre-treated with the AOX inhibitor SHAM, NO emission rates fell in all plant lines (Jayawardhane et al., 2020). However, NO emission rates under hypoxia were highest in overexpressors and lowest in knockdowns, with WT plants responding in a middle ground. It suggests the AOX role in the Pgb1-NO cycle. An increased operation of the Pgb1-NO cycle in the AOX-overexpressing lines is evidenced by NO production and greater expression/activity of Pgb1 and NR, suggesting that AOX can feed the Pgb1-NO cycle, which can contribute to ATP generation under hypoxia (Stoimenova et al., 2007; Vishwakarma et al., 2018). However, further research is needed to determine the difference in ATP/ADP ratios between WT and overexpressors under normoxia.

### A Role for the Phosphorylated Pathway of Serine Biosynthesis in Amino Acid Metabolism Under Hypoxia

In plants and green algae, amino acid metabolism can be supportive of energy metabolism when oxygen is limiting or absent (Reggiani et al., 1988; Rocha et al., 2010; Narsai et al., 2011; Mustroph et al., 2014; Shingaki-Wells et al., 2014; António et al., 2016; Nakamura and Noguchi, 2020; Solhaug et al., 2021). Such amino acid metabolism may reduce lactate and ethanol accumulation, in this way lessening cytosolic acidification (in the case of lactate) and tissue carbon loss (in the case of ethanol). For example, in the green alga *Selenastrum minutum*, there is a stoichiometric decline in aspartate and increases in succinate and alanine at the onset of anoxia (Vanlerberghe et al., 1990). This suggests that aspartate provided the nitrogen for alanine accumulation (with pyruvate providing the carbon skeleton) and the carbon skeleton for succinate accumulation (by a partial reductive tricarboxylic acid cycle). This process continued until the aspartate pool was depleted. However, if inorganic nitrogen was also available for assimilation by glutamine synthase and glutamate-oxoglutarate aminotransferase (the GS-GOGAT cycle), then alanine could continue to accumulate, while maintaining energy and redox balance (Vanlerberghe and Turpin, 1990; Vanlerberghe et al., 1991).

In the current study, we found that high rates of Pgb1-NO cycle activity under hypoxia favored an increased accumulation of amino acids (Figure 1B). This suggests that some of the inorganic nitrogen (nitrate, nitrite) cycling within the Pgb1-NO cycle becomes assimilated by the GS-GOGAT cycle following reduction to ammonium. The increased accumulation of amino acids corresponded to the increased PGK activity, which may indirectly reflect higher glycolytic capacity to provide the necessary carbon skeletons (Figure 1D). Further, the elevated sequestration of carbon and nitrogen into the amino acid pool corresponded with a rapid increased expression of genes encoding each of the enzymes within the PPSB (Figure 3). The pattern of higher gene expression in AOX overexpression lines and decreased expression in AOX knockdowns provides confidence that the transcript regulation patterns observed are likely coordinated. Still transcripts themselves do not provide evidence of a substantial change to metabolism, which might impact upon hypoxia or carbon nitrogen interactions. Therefore, it is worth to compare the data obtained in this study to the profiles of amino acids obtained in the previous investigation (Jayawardhane et al., 2020). They show that in response to hypoxia, the amount of GABA increased in all plant lines, but was much higher in AOX overexpressor. The amount of serine in the knockdown was substantially higher than in WT and overexpressor under normoxia. The level of serine in the knockdown plant lines dropped from the higher values after placing the plants to hypoxic conditions and became identical to the other plant lines. This has a relation to the changes in expression of several genes involved in the serine and GABA biosynthesis observed in this study. The changes in the patterns of several metabolites are linked to the alterations of the transcript regulatory patterns of the enzymes participating in metabolism of these compounds.

Besides generating serine in the chloroplast, the PPSB would also release 2-oxoglutarate that might be necessary to support continued GS-GOGAT activity (Benstein et al., 2013). In *A. thaliana*, the activity of different PGDH isoforms is inhibited by serine but activated by pyruvate-family and aspartate-family amino acids such as alanine, valine, homoserine, homocysteine, and methionine (Okamura and Hirai, 2017; Okamura et al., 2021). This suggests a close integration of the PPSB with other pathways of carbon and nitrogen metabolism, as recently highlighted (Anoman et al., 2015, 2019; Igamberdiev and Kleczkowski, 2018; Watanabe et al., 2021). Under hypoxia, this integration could allow the nitrogen in serine to be readily distributed to other amino acids, such as through transamination reactions.

The increases in transcript amount of the PPSB enzymes was already near maximal by 3 h, the first time point examined following the hypoxic treatment. By comparison, gene transcripts associated with the GABA shunt, a recognized player in low oxygen amino acid metabolism (Bown and Shelp, 1997; Miyashita and Good, 2008; Igamberdiev and Hill, 2018) increased in abundance only at much later time points (Figure 4). In *A. thaliana*, only roots accumulated GABA during a short-term hypoxia treatment, while both roots and shoots accumulated alanine (Mustroph et al., 2014). Hence, the lack of early induction of GABA shunt genes shown here could be a leaf-specific response.

This study identifies the PPSB as an important player in amino acid metabolism under hypoxia, at least when the Pgb1-NO cycle is highly active. In fact, gene transcripts associated with the PPSB enzymes were only induced in the WT and AOX overexpression plants. In the AOX knockdowns, where Pgb1-NO cycle activity was being suppressed, the transcript amount of the PPSB genes actually declined substantially under hypoxia. Serine is positioned at the central node linking the biosynthetic flux from glycolysis to glutathione synthesis and to one-carbon metabolic cycle, which are closely related to the antioxidant capacity (Anoman et al., 2019). Glycine that can be formed from serine, in turn, can generate glyoxylate, which is reduced to glycolate, the latter being an important hypoxic metabolite (Narsai et al., 2013). In the sequence of these reactions, the redox level can be efficiently regulated (Igamberdiev and Kleczkowski, 2018). While the route ending in serine generates NADH, the metabolism beyond that point could oxidize the pool again, e.g., when glycine is produced from serine and glycolate is formed, the whole pathway can efficiently contribute to the redox status during stress (Ho and Saito, 2001; Ros et al., 2014).

Serine serves as a precursor for a variety of essential biomolecules, including nucleic acid bases, phospholipids, and sphingolipids, as well as providing the carbon skeleton for the synthesis of l-tryptophan, l-cysteine and, glutathione. In PPSB-deficient lines, serine lowered the local Cys and GSH pools (Anoman et al., 2019), which, in turn, affects the ascorbate-glutathione cycle. The latter is involved in redox metabolism that results in the oxidation of NADPH to NADP^+^ (Noctor and Foyer, 1998). We can conclude that PPSB is most likely involved in NAD (P)^+^ regeneration in an indirect way. Glycolytic fermentation and the Pgb1-NO cycle both help to regenerate NAD^+^ when oxygen levels are low. In turn, PPSB and the consequent reactions of glycine, glyoxylate and formate metabolism involve NAD turnover, affecting the cellular NAD(P)^+^/NAD(P)H balance and redox status.

Future studies should establish the signal (s) that is controlling PPSB gene expression under hypoxia. It seems unlikely that NO itself is the signal since, even in the AOX knockdowns, NO amounts did increase with hypoxia, while the PPSB gene transcripts declined. On the other hand, a recent study provides evidence that NO is an important signal intermediate controlling the expression of *A. thaliana PGDH1*. *PGDH1* was amongst 23 genes whose increased expression was linked to NO-dependent changes in histone acetylation (Ageeva-Kieferle et al., 2021). Another study showed that a depleted amount of leaf serine could induce the PPSB genes in *A. thaliana* (Modde et al., 2017).

While oxygen deficiency is usually associated with roots and other bulky tissues, it is evident that leaves can also experience this condition, such as during pathogen infection. Interestingly, *A. thaliana* leaf *PGDH1*, *PGDH2*, and *PSAT1* gene expression all increased at the site of *Botrytis cinerea* infection (Benstein et al., 2013), a site recently shown to experience near-anoxic conditions (Valeri et al., 2021). In conclusion, by using AOX transgenic plants as a means to manipulate Pgb1-NO cycle activity, we have identified the PPSB as a previously unrecognized player supporting carbon and nitrogen metabolism under hypoxia.

## Data Availability Statement

The raw data supporting the conclusions of this article will be made available by the authors, without undue reservation.

## Author Contributions

AI, SZ, and GV planned, designed the research, and wrote the manuscript. SZ performed the experiments. SZ and AI analyzed the data. All authors contributed to the article and approved the submitted version.

## Conflict of Interest

The authors declare that the research was conducted in the absence of any commercial or financial relationships that could be construed as a potential conflict of interest.

## Publisher’s Note

All claims expressed in this article are solely those of the authors and do not necessarily represent those of their affiliated organizations, or those of the publisher, the editors and the reviewers. Any product that may be evaluated in this article, or claim that may be made by its manufacturer, is not guaranteed or endorsed by the publisher.
